# Upcycling Waste Bamboo Residues into Mechanically Enhanced TOCNF/Quaternized Cellulose Composite Films

**DOI:** 10.3390/polym18192367

**Published:** 2026-09-28

**Authors:** Wenjun Huang, Wenting Ren, Wenfu Zhang, Xuexia Zhang, Jian Zhang, Ying Zhao

**Affiliations:** 1 Zhejiang Academy of Forestry, Hangzhou 310023, China; 2 College of Material Engineering, Fujian Agriculture and Forestry University, Fuzhou 350108, China

**Keywords:** bamboo residues, steam explosion, quaternized cellulose, TOCNF, electrostatic self-assembly, composite films

## Abstract

Steam explosion processing of bamboo generates substantial quantities of granular residues that are often discarded, leading to resource waste and potential secondary pollution. In this study, a sustainable upcycling strategy was developed to convert these residues into mechanically reinforced cellulose-based composite films. Cellulose isolated from steam-exploded bamboo residues was separately converted into negatively charged TEMPO-oxidized cellulose nanofibrils (TOCNFs) and positively charged quaternized cellulose (QCell), with the latter prepared through modification using 2,3-epoxypropyltrimethylammonium chloride. The oppositely charged cellulose components were subsequently assembled into TOCNF/QCell composite films through electrostatic interactions. At an optimal TOCNF-to-QCell mass ratio of 3:1, the resulting composite film exhibited a tensile strength of 134 MPa, representing an improvement of 23.09% compared with that of the corresponding unmodified TOCNF_1_/Cell_1_ film. SEM observations revealed that the enhanced interfacial interactions facilitated the formation of a dense and compact network structure, thereby contributing to the improved mechanical performance. Although quaternization exerted little influence on the equilibrium moisture uptake of the films, it markedly reduced moisture sorption–desorption hysteresis, indicating enhanced structural reversibility during humidity cycling without substantially altering their overall hydrophilicity. FTIR and XPS analyses confirmed the successful chemical modification of cellulose, while thermogravimetric analysis demonstrated that the thermal stability of the cellulose-based films was largely preserved. Overall, these findings provide a sustainable and effective route for valorizing steam-exploded bamboo residues into high-performance cellulose-based materials.

## 1. Introduction

Steam explosion is an efficient physicochemical pretreatment for the fractionation and structural deconstruction of lignocellulosic biomass and has been widely applied to bamboo fiber separation [1,2]. Rapid depressurization disrupts the hierarchical structure of bamboo tissues, producing long fiber bundles and a substantial granular fraction derived mainly from thin-walled parenchyma cells [3]. Previous studies have focused primarily on fiber separation, morphology, and pressure-induced structural changes, while the fine residues have received limited attention as a feedstock material. Because cellulose can be isolated from both bamboo fibers and parenchyma cells without altering its characteristic crystalline structure, these residues represent a recoverable cellulose resource rather than an unavoidable waste stream. Their valorization could improve the overall material yield and resource efficiency of bamboo processing.

Cellulose films are promising sustainable materials owing to their renewability, low density, biodegradability, and chemical modifiability [4,5]. Their mechanical properties, however, depend strongly on fibril dimensions, structural uniformity, interfacial contact, and the continuity of load-transfer pathways. Multicomponent cellulose systems can address the limitations of single-component films by combining building blocks with complementary dimensions or surface chemistries. Previous studies have shown that interfacial reinforcement and mesoscale network regulation can improve the mechanical performance of cellulose-based films [6,7]. Hydrogen bonding, ionic interactions, and fibrillar entanglement are therefore considered essential for efficient stress transfer and fracture resistance [8].

All-cellulose composites derived from a single waste feedstock provide an integrated alternative to systems containing inorganic fillers, synthetic polymers, or chemically dissimilar biopolymers. TEMPO-mediated oxidation introduces negatively charged carboxylate groups and facilitates cellulose nanofibrillation through electrostatic repulsion [9,10,11], whereas quaternization with EPTAC introduces covalently bound quaternary ammonium groups, imparting a permanent positive charge to cellulose [12]. Previous studies have demonstrated the assembly of oppositely charged cellulose nanofibrils and the formation of cohesive structures through ionic association [13,14]. In the present system, these ionic interactions are expected to act together with hydrogen bonding and fibrillar entanglement, without formation of a new covalently crosslinked network.

In this study, cellulose recovered from steam-exploded bamboo residues was divided into two fractions: one was converted into negatively charged nanoscale TOCNFs, while the other was quaternized into positively charged microscale QCell particles. The two components were recombined at different mass ratios to fabricate all-cellulose composite films, with a non-quaternized TOCNF_1_/Cell_1_ film used as the control. We hypothesized that electrostatic attraction and ionic interactions between TOCNF carboxylate groups and QCell quaternary ammonium groups, together with the dimensional complementarity of nanofibrils and microparticles, would improve interfacial cohesion and network continuity without requiring covalent crosslinking. The effects of composition on optical transmission, fracture morphology, tensile behavior, chemical structure, moisture sorption, thermal transitions, and thermal stability were therefore systematically investigated.

## 2. Materials and Methods

### 2.1. Materials

Four-year-old moso bamboo culms (Phyllostachys edulis (Carr.) H. de Lehaie) were harvested in Hangzhou, Zhejiang Province, China. Sodium chlorite (NaClO_2_, 80%), acetic acid (CH_3_COOH, 99.5%), potassium hydroxide (KOH, 85%), 2,2,6,6-tetramethylpiperidin-1-oxyl (TEMPO), sodium hydroxide (NaOH, 96%), and 2,3-epoxypropyltrimethylammonium chloride (EPTAC) were purchased from Shanghai Macklin Biochemical Co., Ltd. (Shanghai, China). Unless otherwise specified, all reagents were used as received, and deionized water was used throughout the experiments.

### 2.2. Pretreatment of Steam-Exploded Bamboo Residues

Raw bamboo (RB) was subjected to steam explosion in a batch-type apparatus (QBS-80, Hebi Gentle Bioenergy Co., Ltd., Hebi, Henan, China) at 2 MPa for 4 min. The treatment was terminated by instantaneous pressure release, after which the granular fraction was collected from the reactor. The steam-exploded bamboo residues (SEBR) were purified using a procedure adapted from a previously reported method for isolating cellulose from bamboo tissues. Briefly, the residues were treated with a 1 wt% sodium chlorite solution at 75 °C for 1 h, with the pH adjusted to 4.0 using glacial acetic acid. This delignification step was repeated four times to promote the removal of residual lignin. The delignified material was subsequently treated with a 5 wt% KOH solution at 90 °C for 2 h to remove hemicellulosic components. The resulting cellulose-rich particles were thoroughly washed with deionized water until the washing effluent reached neutral pH and were then dried at 60 °C.

### 2.3. Preparation of the Quaternized Cellulose

The purified cellulose particles were dispersed in a 5 wt% aqueous NaOH solution at a solid loading of 2% (*w*/*v*). EPTAC was then added at a molar ratio of 1:1 relative to the anhydroglucose units of cellulose. The quaternization reaction was conducted at 60 °C for 8 h under continuous stirring. After reaction, the product was repeatedly washed with deionized water until neutral to remove residual NaOH and unreacted EPTAC. The resulting positively charged quaternized cellulose was designated QCell. Before subsequent blending, the QCell suspension was ultrasonicated at 500 W for 40 min to reduce particle agglomeration and improve dispersion.

### 2.4. Preparation of TEMPO-Oxidized Cellulose Nanofibrils

Purified cellulose (1.0 g) was dispersed in deionized water (100 mL) containing TEMPO (0.1 mmol) and sodium bromide (NaBr, 1 mmol). Oxidation was initiated by the gradual addition of sodium hypochlorite (NaClO, 5 mmol) under continuous stirring at room temperature. During oxidation, the suspension pH was maintained at approximately 10.5 by periodic adjustment. The reaction was considered complete when the pH remained stable without further adjustment, after which the suspension was adjusted to pH 7.0 using 0.5 M HCl. The TEMPO-oxidized cellulose was thoroughly washed with deionized water to remove residual reagents and stored at 4 °C. The oxidized-cellulose dispersion was subsequently processed using a high-pressure homogenizer to induce nanofibrillation, yielding a TOCNF suspension with a concentration of approximately 1.27 wt%.

### 2.5. Preparation of TOCNF/QCell Composite Films

TOCNF and QCell suspensions were mixed at dry mass ratios of 1:1, 2:1, and 3:1, with a total dry mass of 110 mg for each formulation. QCell was added to the corresponding TOCNF suspension, and the total volume was adjusted to 50 mL with deionized water, resulting in a solids concentration of 2.2 mg mL^−1^ (0.22%, *w*/*v*). The mixtures were magnetically stirred at 600 rpm for 2 h and subsequently ultrasonicated at 600 W for 40 min. Each dispersion was vacuum-filtered through a hydrophilic PTFE membrane with a diameter of 50 mm and a pore size of 0.22 μm. The resulting wet films were dried in a vacuum oven at 80 °C and −0.1 MPa for 4 h without additional hot pressing, yielding films with a thickness of approximately 0.06 mm. Before characterization, the films were conditioned under ambient laboratory conditions for 2 days. According to the dry mass ratios of TOCNF to QCell, the films were designated TOCNF1/QCell1, TOCNF_2_/QCell_1_, and TOCNF_3_/QCell_1_, respectively. The TOCNF_1_/Cell_1_ control film was prepared by replacing QCell with an equal dry mass of unmodified cellulose following the same procedure.

### 2.6. Characterization of TOCNF/QCell Composite Films

The visual appearance and contact transparency of the films were documented by digital photography. The optical transmittance of the films was measured in transmittance mode using a UV–Vis–NIR spectrophotometer (UV-3600i Plus, Shimadzu, Kyoto, Japan) over the wavelength range of 200–800 nm. The results are presented as transmittance spectra. The fracture-surface morphology and elemental distribution of the films were examined by scanning electron microscopy (SEM; S-3400N, Hitachi, Tokyo, Japan). The tensile properties of the films were measured using a TA.XTplus Texture Analyser (Stable Micro Systems, Godalming, Surrey, UK) at a crosshead speed of 2 mm min^−1^. The film specimens were cut into strips approximately 3 mm in width, with an initial grip separation of 20 mm. Ten specimens were tested for each film formulation, and the results were expressed as the mean ± standard deviation.

The zeta potentials of the QCell and TOCNF dispersions were measured using a Zetasizer Nano ZS90 instrument (Malvern Instruments Ltd., Malvern, UK). The samples were dispersed in deionized water and measured in triplicate. The results were expressed as the mean ± standard deviation. Fourier transform infrared spectra (FTIR, IRTracer-100, Shimadzu, Kyoto, Japan) were recorded in attenuated total reflectance (ATR) mode over the wavenumber range of 4000–400 cm^−1^ at a resolution of 4 cm^−1^. Before comparison, all spectra were subjected to baseline correction and min–max normalization using the same processing parameters. The normalized absorbance was calculated as A_norm_= (A − A_min_)/ (A_max_ − A_min_). X-ray photoelectron spectroscopy (XPS; K-Alpha, Thermo Fisher Scientific, Waltham, MA, USA) was used to determine the surface elemental composition and chemical states of the films. X-ray diffraction patterns were recorded using an X-ray diffractometer (SmartLab SE, Rigaku, Tokyo, Japan) over a 2θ range of 10–80° at a scanning rate of 2° min^−1^.

Thermogravimetric (TG) and derivative thermogravimetric (DTG) analyses were conducted using a thermogravimetric analyzer (TGA2, Mettler Toledo, Zurich, Switzerland) under a nitrogen atmosphere. Approximately 5 mg of each dried film was heated from 25 to 600 °C at a rate of 10 °C min^−1^ under a nitrogen flow of 50 mL min^−1^. Differential scanning calorimetry (DSC; Discovery DSC 2500, TA Instruments, New Castle, DE, USA) was performed using standard aluminum pans under a nitrogen atmosphere. Approximately 1.8–6.8 mg of each film sample was equilibrated at 40 °C and subsequently heated to 200 °C at a heating rate of 10 °C min^−1^.

The moisture sorption behavior of the films was evaluated using a dynamic vapor sorption analyzer (DVS Intrinsic, Surface Measurement Systems, London, UK). Approximately 30 mg of each film specimen was initially conditioned at 0% relative humidity (RH) to remove residual moisture. The RH was then increased stepwise from 0% to 90% in 10% increments and subsequently from 90% to 95% in a 5% increment, followed by desorption to 0% RH using the reverse sequence. Equilibrium at each RH level was considered to be reached when the rate of mass change was below 0.02% min^−1^.

ChatGPT 5.5 (OpenAI) was used to assist in preparing the schematic diagram of the film fabrication process. The authors reviewed and edited the diagram to ensure that it accurately represents the experimental procedure.

## 3. Results and Discussion

### 3.1. Effects of Steam Explosion on Bamboo Residues

The SEM images show that RB consisted mainly of short, irregular particles and fragments, whereas SEBR exhibited more clearly defined elongated fibrous structures, more pronounced separation between fibrous fragments, and localized longitudinal grooves and surface damage. These morphological changes indicate that steam explosion promoted the disintegration of the original bamboo tissue structure. Based on the mechanism of steam explosion, these changes can be attributed primarily to steam penetration under high pressure and the thermomechanical effects generated by rapid depressurization. Similar changes in the particle morphology and structural organization of lignocellulosic biomass after steam explosion were also reported by Figel et al. [15]. As shown in Figure 1c, both RB and SEBR exhibited the characteristic FTIR bands of bamboo-derived lignocellulosic powders. The bands at approximately 3400 and 2904 cm^−1^ were assigned to O–H and C–H stretching vibrations, respectively, whereas those at 1734, 1600, and 1241 cm^−1^ were mainly associated with hemicellulose- and lignin-related structures. Changes in these bands after steam explosion were consistent with the partial degradation of hemicellulose and structural rearrangement of lignin. However, no new characteristic bands were detected, suggesting that the cellulose backbone was largely preserved. The XRD patterns (Figure 1d) exhibited the characteristic reflections of cellulose I at approximately 16°, 22°, and 34°. The absence of noticeable peak shifts indicated that steam explosion modified the noncrystalline lignocellulosic matrix without causing a detectable transformation of the cellulose crystalline allomorph [16,17]. The TG and DTG curves (Figure 1e,f) further demonstrated the differences in thermal degradation behavior between RB and SEBR. The initial decomposition temperature decreased from 202.9 °C for RB to 173 °C for SEBR, indicating the formation or increased exposure of thermally less stable components following hemicellulose hydrolysis and polysaccharide depolymerization. In contrast, the principal DTG peak shifted from 337.9 to 351.9 °C, accompanied by a more pronounced low-temperature shoulder for SEBR. These changes indicate a clearer distinction between the degradation of thermally less stable noncellulosic components and that of the remaining cellulose-rich fraction. Overall, steam explosion partially deconstructed the lignocellulosic matrix while preserving the cellulose I structure and shifting the principal decomposition event toward a higher temperature.

### 3.2. Fabrication Strategy and Surface-Charge Characteristics of TOCNF/QCell Composite Films

The zeta-potential measurements confirmed the charge complementarity between the two cellulose components (Figure 2). QCell exhibited positive zeta-potential values of approximately +15.80 to +17.45 mV, whereas TOCNF showed strongly negative values ranging from approximately −52.54 to −46.38 mV. The positive potential of QCell was attributed to the quaternary ammonium groups introduced by EPTAC, whereas the negative potential of TOCNF originated from the surface carboxylate groups generated during TEMPO-mediated oxidation. The opposite surface charges favored electrostatic association between QCell and TOCNF during film formation.

The overall preparation strategy is illustrated in Figure 3. A cellulose-rich fraction was first recovered from the parenchyma-rich granular residues of steam-exploded bamboo through sequential delignification and alkali treatment. The purified cellulose was then divided into two fractions with distinct dimensions and surface chemistries. One fraction was subjected to TEMPO-mediated oxidation and mechanical fibrillation to produce negatively charged nanoscale TOCNFs. The other fraction was etherified with EPTAC to introduce permanent quaternary ammonium groups while largely retaining its micrometer-scale particulate morphology, thereby producing positively charged QCell. This division-and-recombination strategy generated two complementary cellulose components from a single waste feedstock: TOCNF served primarily as the continuous reinforcing phase, whereas QCell provided dispersed cationic domains for interfacial association.

When the two components were mixed, the carboxylate groups on TOCNF interacted electrostatically with the quaternary ammonium groups on QCell, forming noncovalent ion pairs at their interfaces. Meanwhile, the high-aspect-ratio TOCNFs surrounded and bridged the larger QCell particles, establishing continuous physical connections between neighboring domains. During drying, the decreasing distance between cellulose surfaces increased interfacial contact, while the remaining hydroxyl groups contributed additional hydrogen-bonding interactions. Consequently, the final film network was stabilized through the combined effects of electrostatic association, hydrogen bonding, fibrillar entanglement, and drying-induced densification rather than covalent crosslinking.

The different dimensions of TOCNF and QCell determined their respective structural roles and made the film properties dependent on their mass ratio. At a low TOCNF content, the fibrillar framework was insufficiently continuous, leaving gaps between the larger QCell particles. Increasing the TOCNF fraction improved the continuity of the nanoscale network and enhanced load transfer across adjacent QCell domains, while sufficient QCell remained available to provide oppositely charged interfacial sites. This balance was most effectively achieved at a TOCNF-to-QCell mass ratio of 3:1. Consistent with the proposed assembly mechanism, the TOCNF_3_/QCell_1_ film exhibited the most compact cross-sectional morphology and the highest tensile strength among the investigated formulations. These results demonstrate that controlled differentiation and recombination of cellulose derived from a single bamboo-residue feedstock enabled the construction of a mechanically reinforced multiscale network.

### 3.3. Optical Properties and Fracture Morphology of TOCNF/QCell Composite Films

The optical properties of the films were evaluated by digital photography and UV–Vis spectroscopy. As shown in Figure 4, the TOCNF_1_/Cell_1_ control and TOCNF_1_/QCell_1_ films exhibited relatively similar transmittance in the visible-light region. In contrast, the transmittance decreased as the TOCNF-to-QCell mass ratio increased from 1:1 to 3:1. At 650 nm, the TOCNF_2_/QCell_1_ and TOCNF_3_/QCell_1_ films showed lower transmittance than the control film. This decrease may be related to enhanced internal light scattering caused by composition-dependent changes in fibril aggregation, interfacial density, and structural heterogeneity.

Cross-sectional SEM images provide direct morphological support for this interpretation (Figure 5). All films displayed lamellar structures formed by deposition and consolidation during drying. The TOCNF_1_/Cell_1_ control contained more visible structural discontinuities, whereas the TOCNF/QCell films showed more compact and continuous cross-sections. Within the TOCNF/QCell series, increasing the TOCNF fraction reduced the visible gaps and produced the most uniform cross-section at a TOCNF-to-QCell mass ratio of 3:1. This morphology follows the assembly mechanism reported for oppositely charged cellulose-based colloids: electrostatic association suppresses interfacial separation, and nanofibrils bridge adjacent dispersed domains. The SEM result therefore demonstrates that QCell quaternization improved interfacial integration and that sufficient TOCNF was required to establish a continuous fibrillar framework. Because visible voids and poorly connected interfaces act as stress-concentration sites, the compact 3:1 morphology provides the structural explanation for the maximum tensile strength measured for the same composition.

### 3.4. Tensile Performance of TOCNF/QCell Composite Films

The tensile properties are summarized in Figure 6a–e. The non-quaternized TOCNF_1_/Cell_1_ control exhibited an average tensile strength of 108.87 ± 11.82 MPa. Incorporation of QCell increased the tensile strength, and the TOCNF_3_/QCell_1_ film reached approximately 134 MPa, representing a 23.09% improvement over the control. The tensile toughness increased from approximately 0.58 to 1.20 MJ m^−3^ across the composition series, whereas Young’s modulus varied non-monotonically, indicating that the strength enhancement did not arise simply from increased stiffness. The elongation at break remained within approximately 0.8–1.3%, although relatively large variations were observed for some formulations. The coincidence of the highest tensile strength with the most compact cross-sectional morphology indicates a clear composition–structure–property relationship rather than a strength change caused solely by chemical modification. The representative stress–strain curves (Figure 6e) further show that the 1:1 film sustained greater deformation but lower stress, whereas increasing the TOCNF fraction enhanced the tensile strength and restricted deformation at failure. Ionic interactions between the negatively charged carboxylate groups of TOCNF and the positively charged quaternary ammonium groups of QCell can increase resistance to interfacial separation, while hydrogen-bonding interactions and fibril entanglement facilitate stress transfer throughout the interconnected network. The 3:1 formulation provided sufficient TOCNF to bridge adjacent QCell domains while retaining interfacial quaternary ammonium sites, thereby achieving the best balance of mechanical properties among the investigated formulations. Therefore, the optimal TOCNF-to-QCell mass ratio determined in this study was 3:1. As shown in Figure 6f, the TOCNF_3_/QCell_1_ film exhibited a tensile strength of 134 MPa, exceeding those of the selected cellulose-based films reported in the literature (approximately 29–118 MPa) [18,19,20,21,22,23,24,25,26]. This improvement is attributed to ionic interactions, hydrogen bonding, and fibril entanglement within the compact network. These results demonstrate the mechanical advantage of the proposed all-cellulose film prepared from a single bamboo-waste source.

### 3.5. Chemical Structural Analysis of TOCNF/QCell Composite Films

FTIR spectra of the control and TOCNF/QCell films are compared in Figure 7a. All samples retained the characteristic cellulose absorption envelope, including the broad O–H stretching band near 3315 cm^−1^, C–H stretching near 2906–2916 cm^−1^, absorption near 1600 cm^−1^ associated with carboxylate groups and absorbed water, and cellulose skeletal/C–O–C vibrations near 987 cm^−1^. These assignments agree with the reported spectra of TEMPO-oxidized and cationized cellulose. The persistence of the cellulose fingerprint bands confirms that neither modification route destroyed the polysaccharide backbone. Changes in O–H-band width and symmetry across the mass-ratio series demonstrate that the distribution of hydroxyl-group environments changed after QCell incorporation and composition adjustment. Because no new diagnostic covalent-bond band or systematic large peak shift was observed, the FTIR data support rearrangement of hydrogen-bonded environments but do not support covalent crosslinking between TOCNF and QCell. The spectroscopic result is therefore consistent with the proposed noncovalent electrostatic-assembly mechanism.

The XRD patterns (Figure 7b) contained broad diffraction features near 2θ = 15.4° and 22.4°, corresponding to characteristic cellulose-I reflections. All formulations retained the same principal peak positions, demonstrating that quaternization, TEMPO oxidation, blending, and drying did not produce a detectable transformation of the cellulose-I allomorph. TEMPO oxidation under controlled conditions has likewise been reported to preserve the cellulose crystal structure while modifying accessible surface hydroxyl groups. Variations in peak breadth and relative intensity among the present films indicate differences in fibril organization and orientation, but quantitative crystallinity changes cannot be assigned without validated peak deconvolution. The defensible result is that the 23.09% strength increase occurred without formation of a new crystalline phase. Accordingly, the mechanical improvement originates primarily from interfacial association and network densification observed by SEM rather than from a crystal-allomorph transition.

XPS provided direct evidence for QCell surface functionalization (Figure 7c–e). Survey spectra contained the dominant C 1s and O 1s signals of cellulose, while an N 1s signal appeared in the quaternized samples. High-resolution C 1s components near 284.8, 286.4, and 289.2 eV were assigned to C–C/C–H, C–O, and O–C=O environments, respectively. The N 1s region contained a component near 402 eV assigned to positively charged quaternary ammonium nitrogen (R_4_N^+^), consistent with XPS analyses of quaternary-ammonium-modified cellulose [27]. Together with the positive zeta potential of QCell and the negative potential of TOCNF, these results support electrostatic attraction and electrostatic association between the two cellulose components.

### 3.6. Moisture Sorption and Thermal Stability of TOCNF/QCell Composite Films

The DVS results distinguish equilibrium moisture uptake from sorption reversibility (Figure 8a,b). Both films exhibited nonlinear increases in moisture uptake with increasing RH, particularly at high humidity, owing to water binding at accessible polar sites followed by cooperative water–water interactions and cellulose swelling [28]. Their similar equilibrium moisture contents indicate that QCell incorporation did not substantially alter the overall hygroscopicity of the films. However, the TOCNF/QCell film exhibited a smaller sorption–desorption hysteresis loop, suggesting that ionic interactions between TOCNF and QCell restricted irreversible structural rearrangement during humidity changes and improved moisture-response reversibility. Nevertheless, long-term cyclic stability requires further verification through repeated humidity cycling. The TG and DTG results are shown in Figure 8c,d, and the corresponding thermal parameters are summarized in Table 1. The initial mass loss was associated with the removal of physically adsorbed water, whereas the major mass loss between approximately 200 and 400 °C resulted from cellulose dehydration and backbone decomposition. As the TOCNF/QCell ratio increased from 1:1 to 3:1, T_5_% decreased from 224.8 to 212.8 °C and T_onset decreased from 284.9 to 270.5 °C, indicating a moderate reduction in initial thermal stability. In contrast, the relatively small changes in T_50_% and T_max suggest that the principal cellulose-degradation stage remained largely unchanged.

The residual mass at 600 °C remained nearly constant at 29.0–29.7% for all films, indicating that the TOCNF/QCell ratio had little influence on the final char yield. Although the introduction of quaternary ammonium and carboxylate groups moderately affected the initial degradation temperatures, the relatively small variations in T_max and residual mass demonstrate that neither quaternization nor TEMPO oxidation substantially altered the principal thermal-degradation pathway of cellulose. Therefore, the incorporation of QCell and TOCNF caused no evident adverse effects on the main thermal decomposition behavior or char-forming ability of the cellulose-based films.

DSC provided further information on the thermal behavior of the films (Figure 8e–h). The TOCNF_1_/Cell_1_ and TOCNF_1_/QCell_1_ films exhibited endothermic peaks at approximately 178.1 and 176.8 °C, respectively, whereas the peaks of the TOCNF_2_/QCell_1_ and TOCNF_3_/QCell_1_ films shifted to approximately 199 °C. This shift indicates composition-dependent changes in the thermal environment of the cellulose chains and suggests stronger intermolecular interactions and improved compatibility at higher TOCNF contents.

## 4. Conclusions

This study developed a strategy for valorizing granular residues from steam-exploded bamboo by converting recovered cellulose into negatively charged TOCNFs and positively charged QCell. Zeta-potential measurements confirmed the charge complementarity of the two components, while XPS verified the introduction of quaternary ammonium groups. Electrostatic association between TOCNF carboxylate groups and QCell quaternary ammonium groups, together with hydrogen bonding, fibrillar entanglement, and drying-induced densification, promoted the formation of a compact multiscale network. The optimal TOCNF_3_/QCell_1_ film achieved a tensile strength of 134 MPa, representing a 23.09% improvement over the TOCNF_1_/Cell_1_ control, although its visible-light transmittance was lower than that of the control. TGA showed that the principal thermal-degradation stage was largely preserved, whereas DSC revealed composition-dependent thermal events that shifted from approximately 177–178 °C to approximately 199 °C at higher TOCNF/QCell ratios. QCell incorporation had little effect on equilibrium moisture uptake but reduced sorption-desorption hysteresis. Overall, this work demonstrates a water-based, all-cellulose route integrating residue-derived cellulose valorization, multiscale network construction, and electrostatic assembly to enhance film performance.

## Figures and Tables

**Figure 1:**
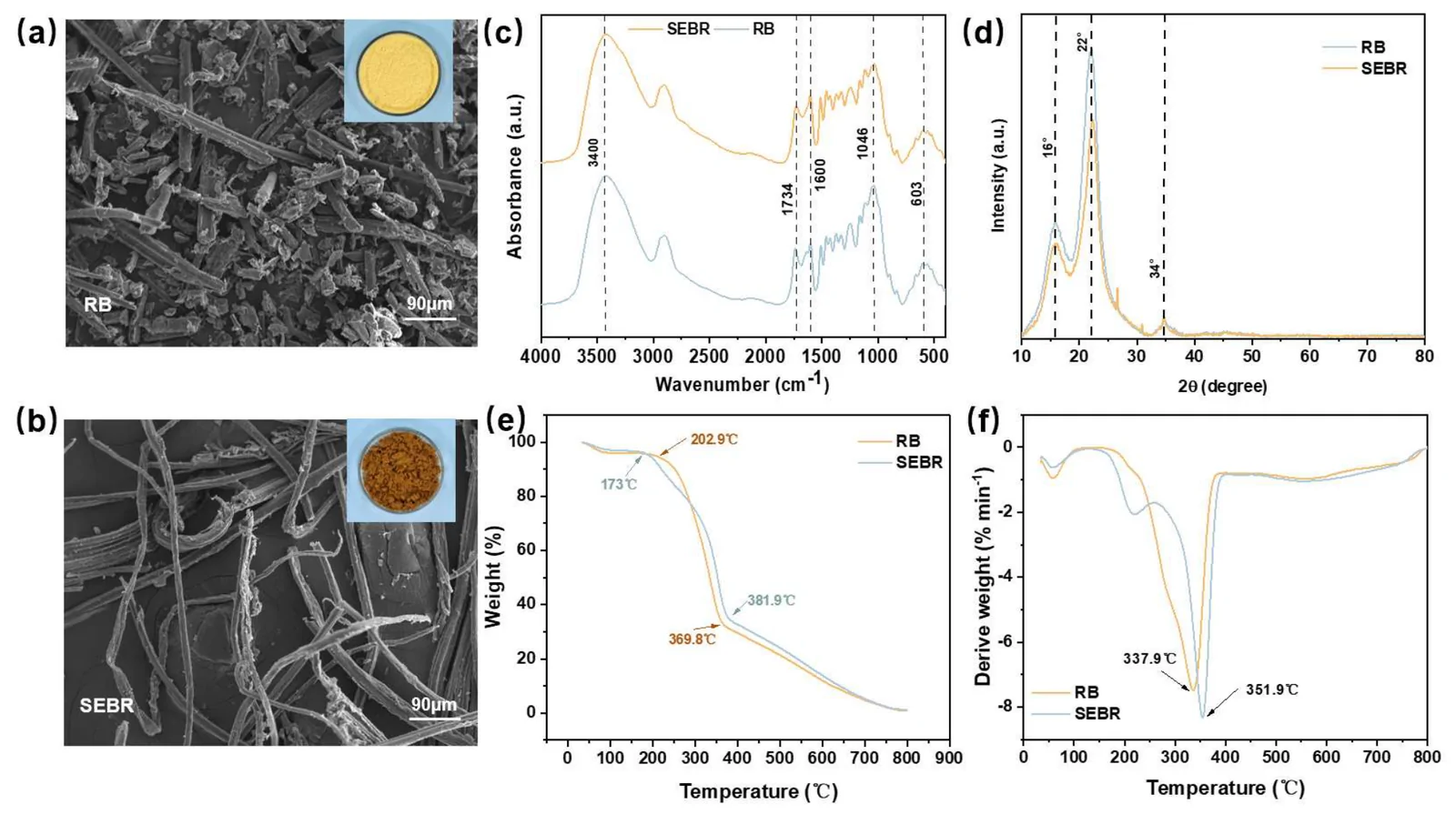
Structural and physicochemical changes in bamboo induced by steam explosion: SEM images of (**a**) raw bamboo powder (RB) and (**b**) steam-exploded bamboo residues (SEBR); (**c**) FTIR spectra; (**d**) XRD patterns; (**e**) TG curves; and (**f**) DTG curves of RB and SEBR.

**Figure 2:**
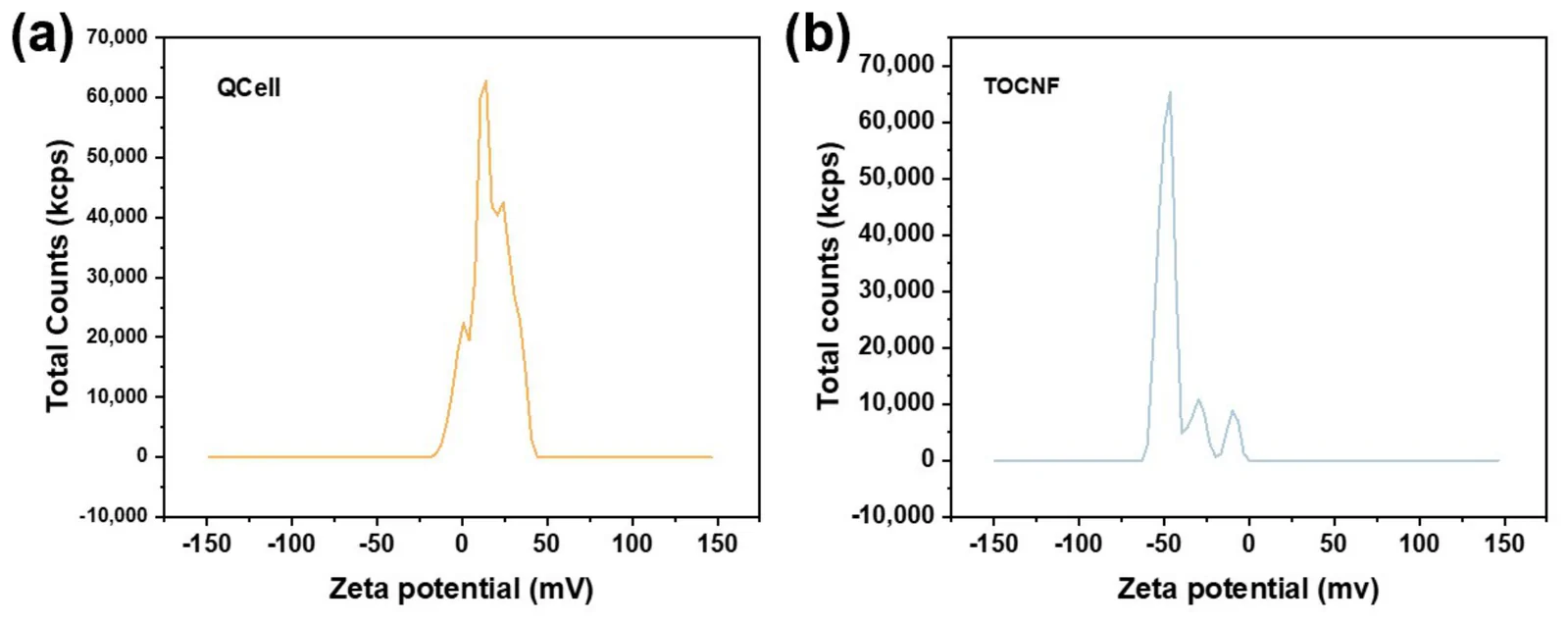
Representative zeta-potential distributions of aqueous dispersions of (**a**) QCell and (**b**) TOCNF.

**Figure 3:**
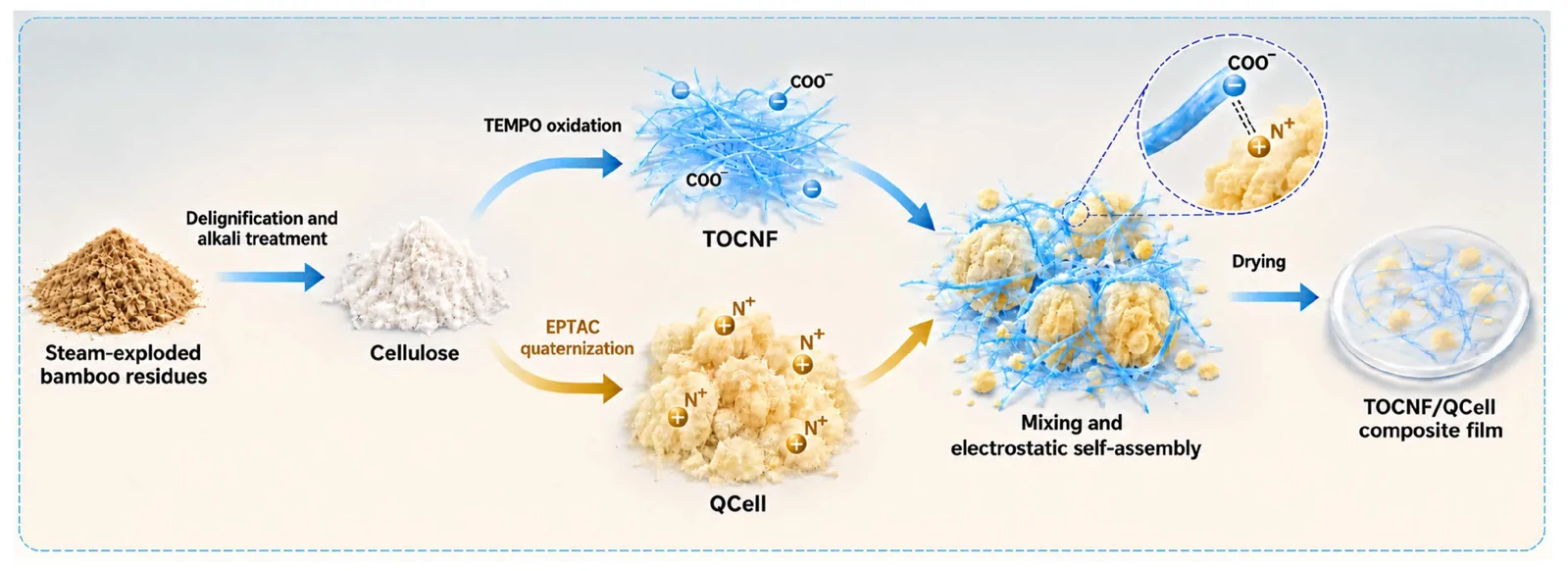
Schematic illustration of the preparation and assembly of TOCNF/QCell composite films.

**Figure 4:**
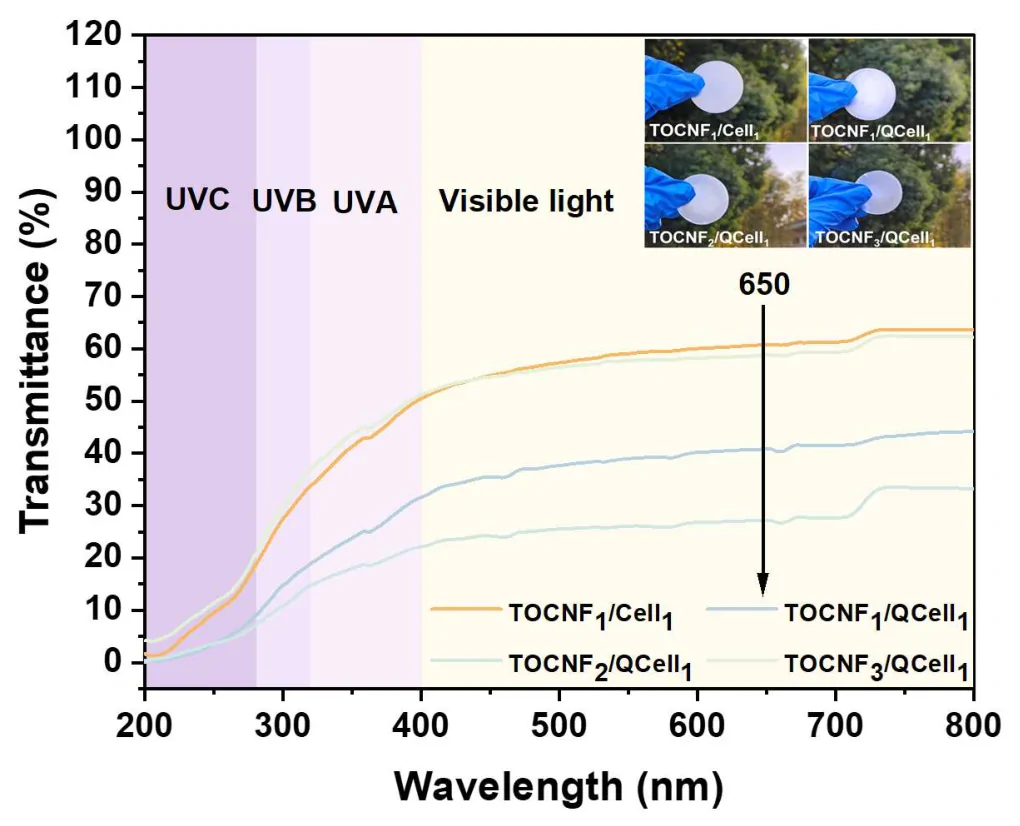
UV–Vis transmittance spectra of the TOCNF/QCell composite films.

**Figure 5:**
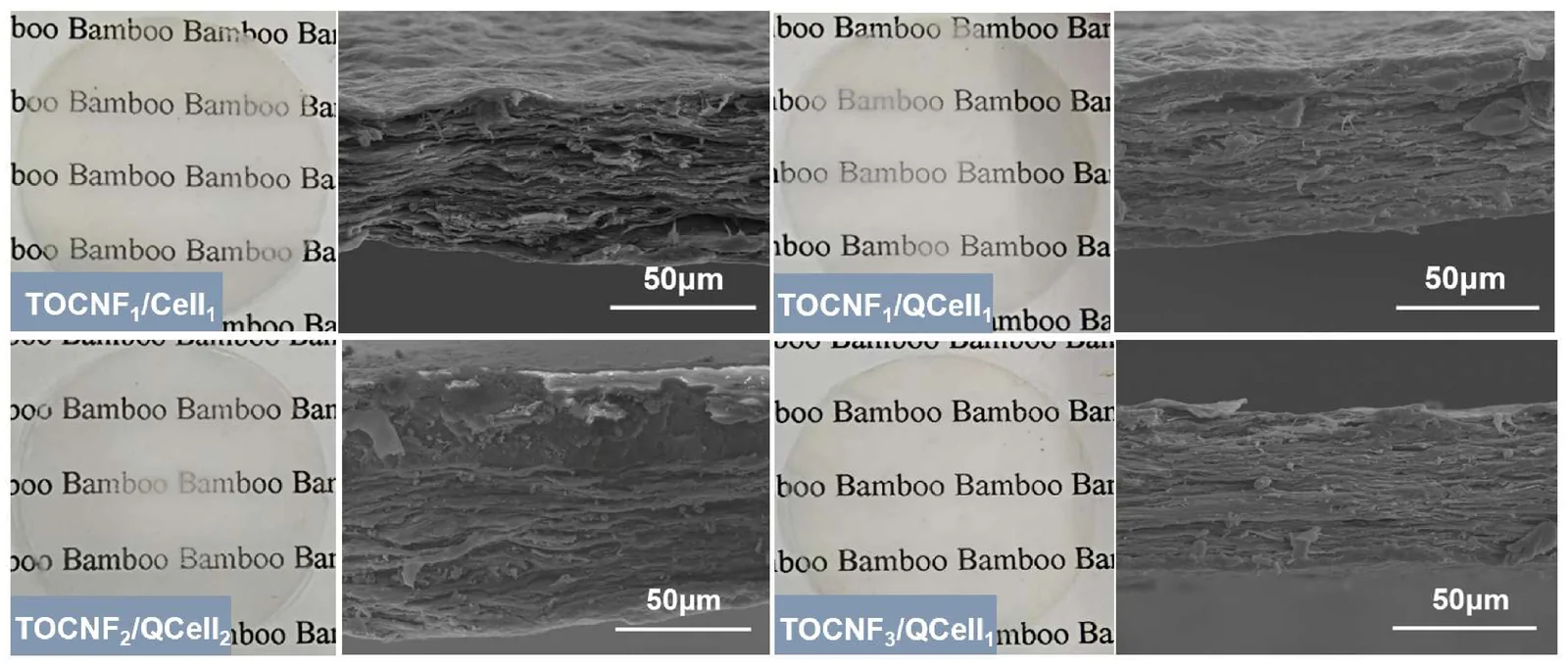
Digital photographs of the films placed over a printed background and cross-sectional SEM images showing the microstructure of the composite films.

**Figure 6:**
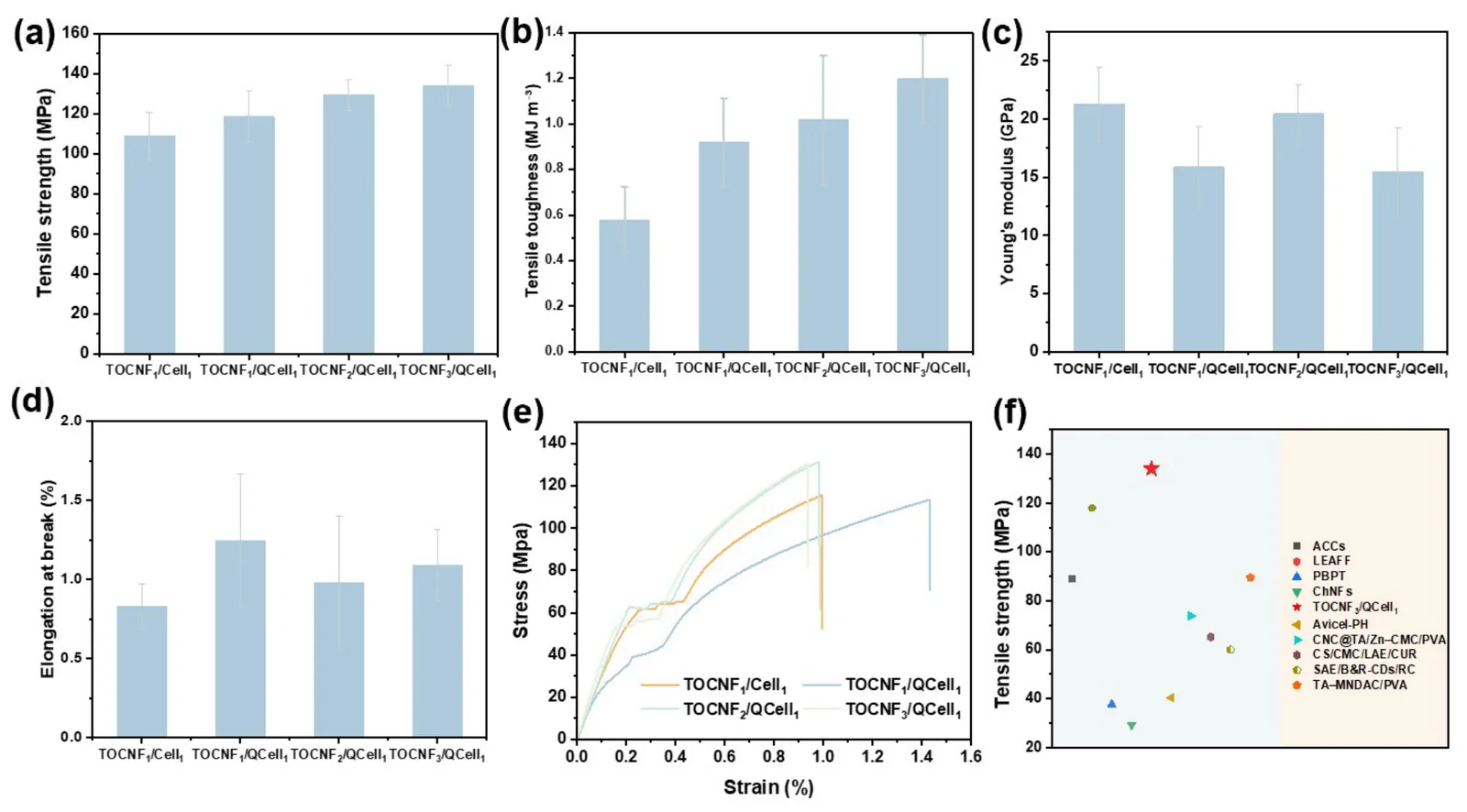
Mechanical properties of the composite films: (**a**) tensile strength, (**b**) tensile toughness, (**c**) Young’s modulus, (**d**) elongation at break, and (**e**) representative stress–strain curves; (**f**) comparison of the tensile strength of the optimal TOCNF_3_/QCell_1_ film with those of recently reported cellulose-based films.

**Figure 7:**
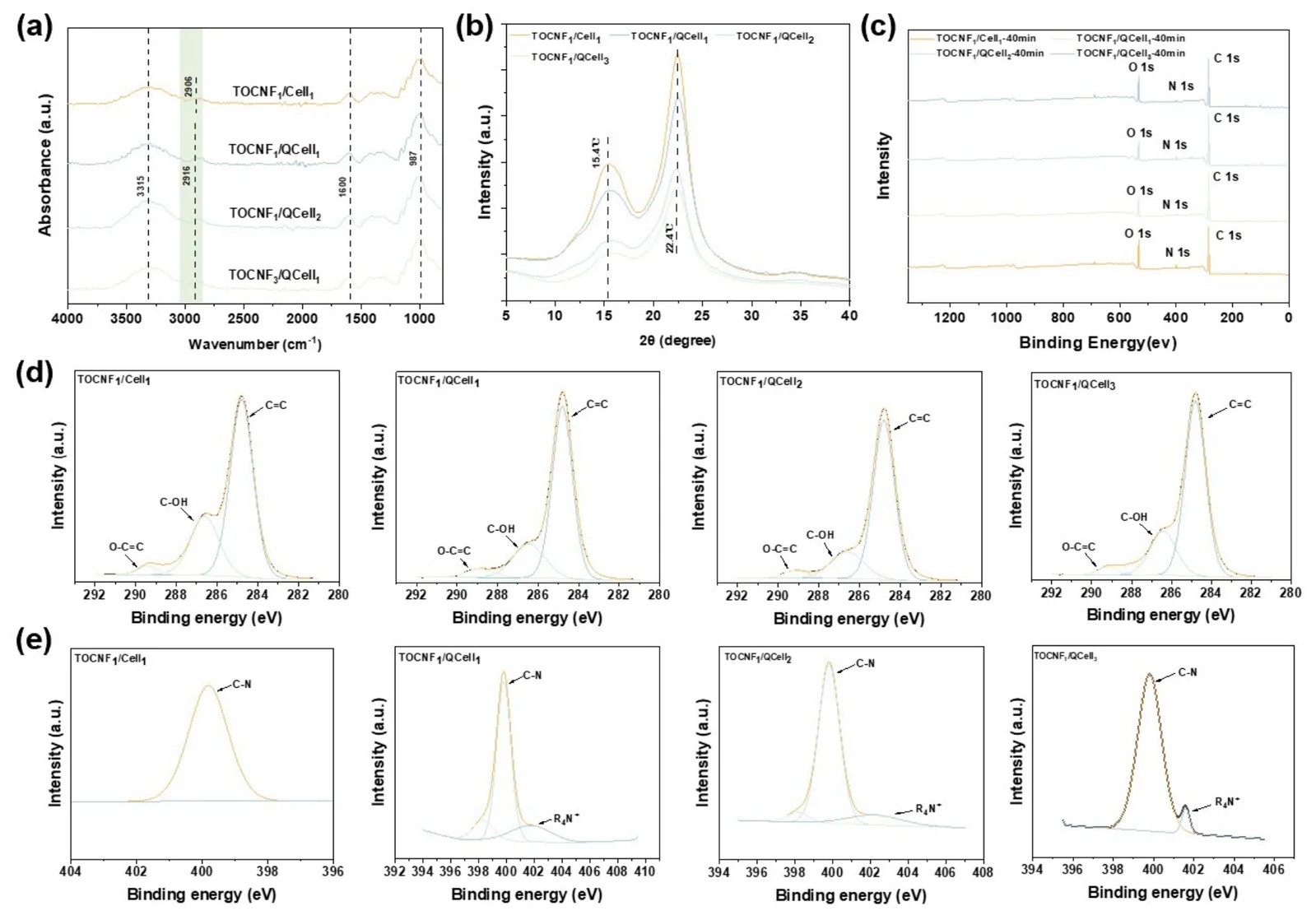
Structural and chemical characterization of the composite films: (**a**) FTIR spectra; (**b**) XRD patterns; (**c**) XPS survey spectra; (**d**) high-resolution C 1s spectra; and (**e**) high-resolution N 1s spectra.

**Figure 8:**
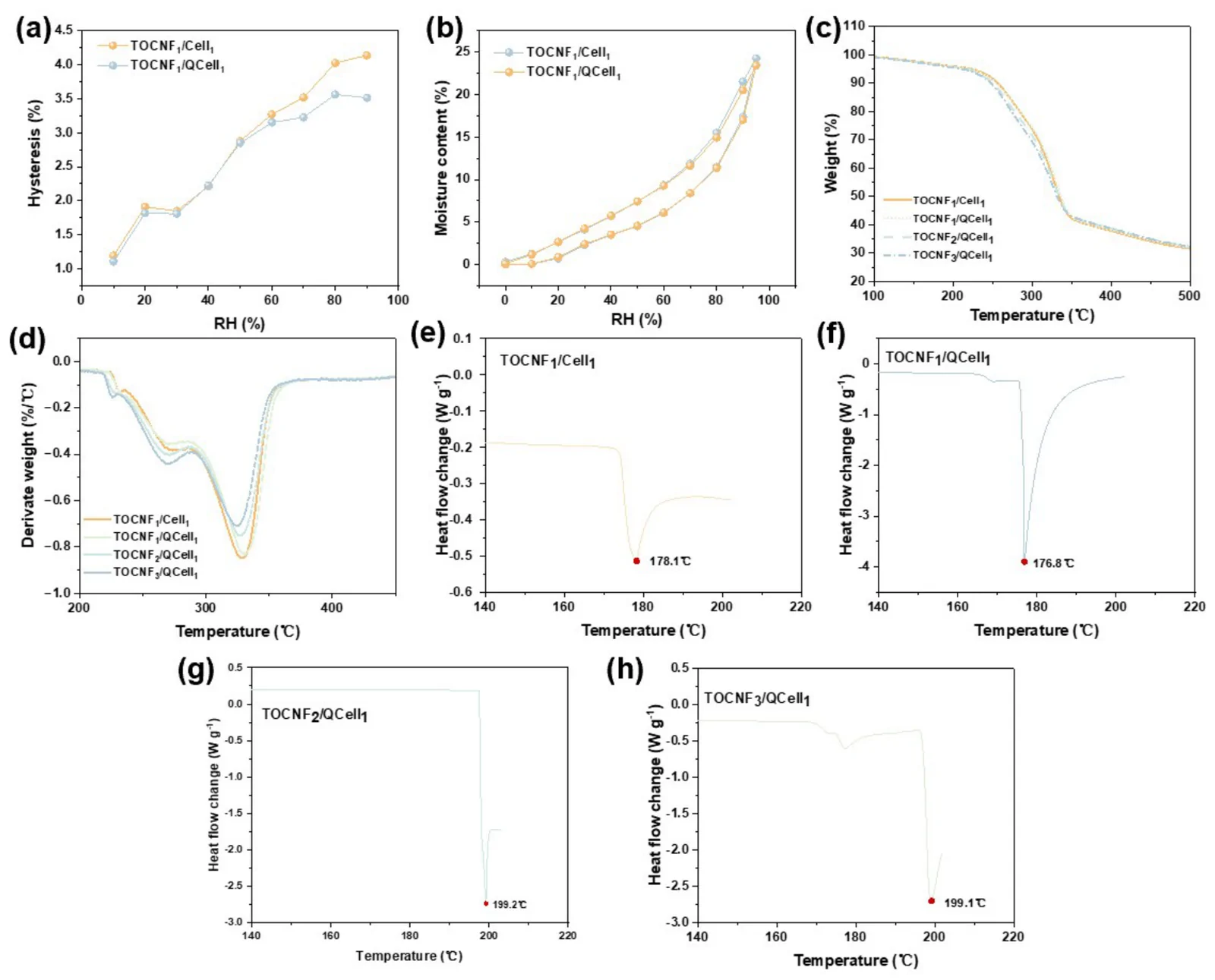
Moisture sorption and thermal behavior of the composite films: (**a**) sorption–desorption hysteresis; (**b**) moisture sorption isotherms; (**c**) TG curves; (**d**) DTG curves; and (**e**–**h**) DSC heat-flow curves.

**Table 1:** Quantitative thermogravimetric parameters of the TOCNF/QCell composite films.

Sample	T_5_% (°C)	T_10_% (°C)	T_50_% (°C)	T_onset (°C)	T_max (°C)	Residue at 600 °C (%)
**TOCNF_1_/Cell_1_**	**225.6**	**256.6**	**334.5**	**283.1**	**328.5**	**29.0**
**TOCNF_1_/QCell_1_**	**224.8**	**254.8**	**337.1**	**284.9**	**331.0**	**29.4**
**TOCNF_2_/QCell_1_**	**217.2**	**250.4**	**334.4**	**276.5**	**327.5**	**29.7**
**TOCNF_3_/QCell_1_**	**212.8**	**248.1**	**331.7**	**270.5**	**324.5**	**29.6**

## Data Availability

The original contributions presented in this study are included in the article. Further inquiries can be directed to the corresponding authors.
